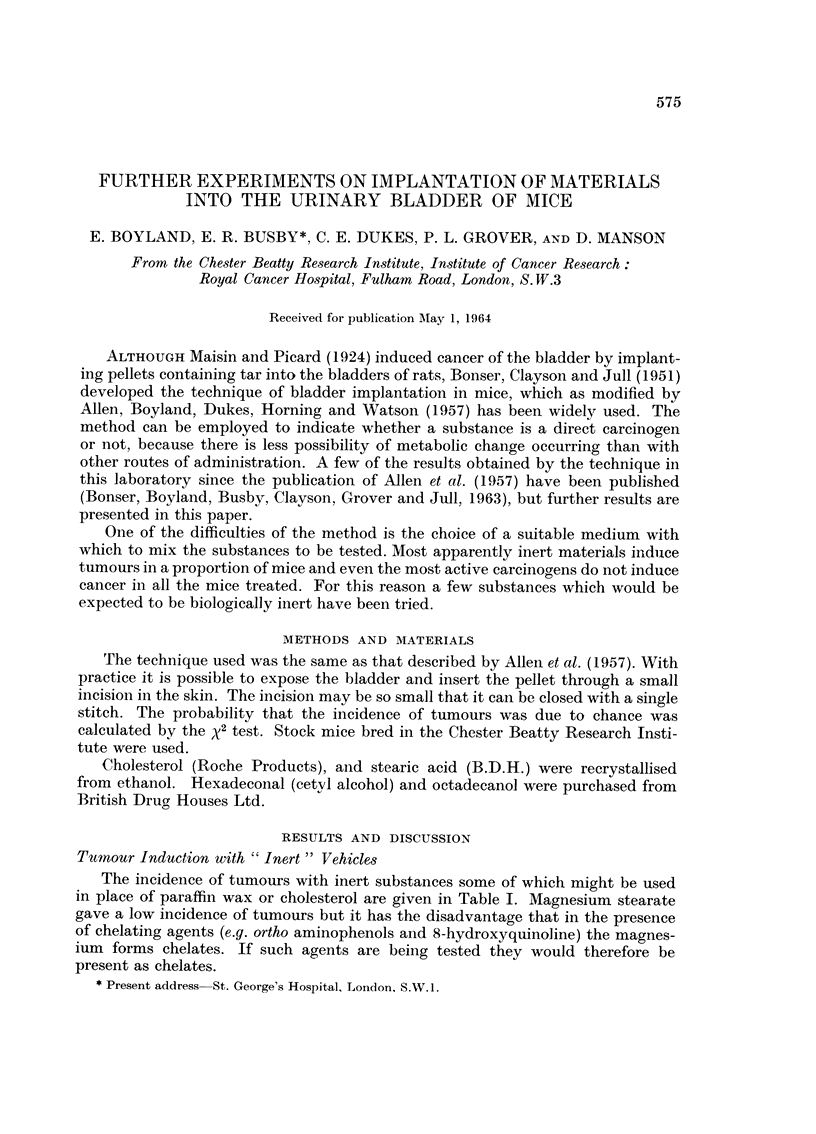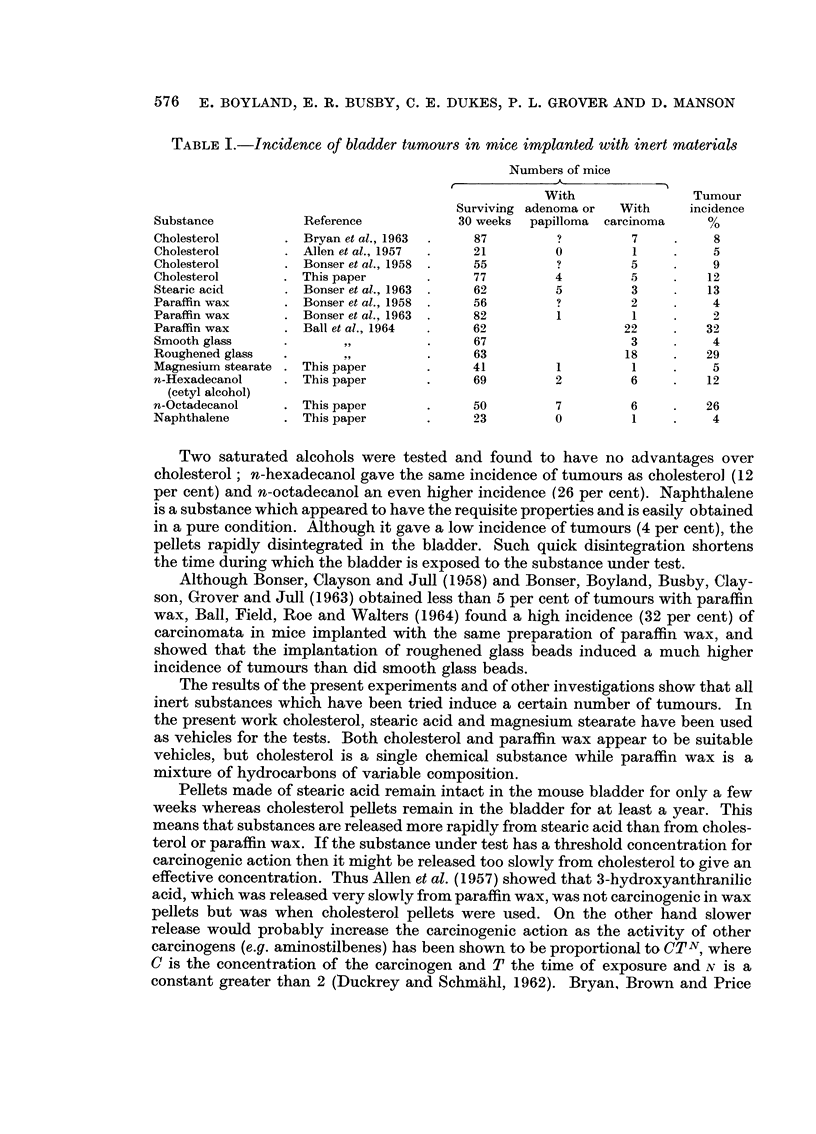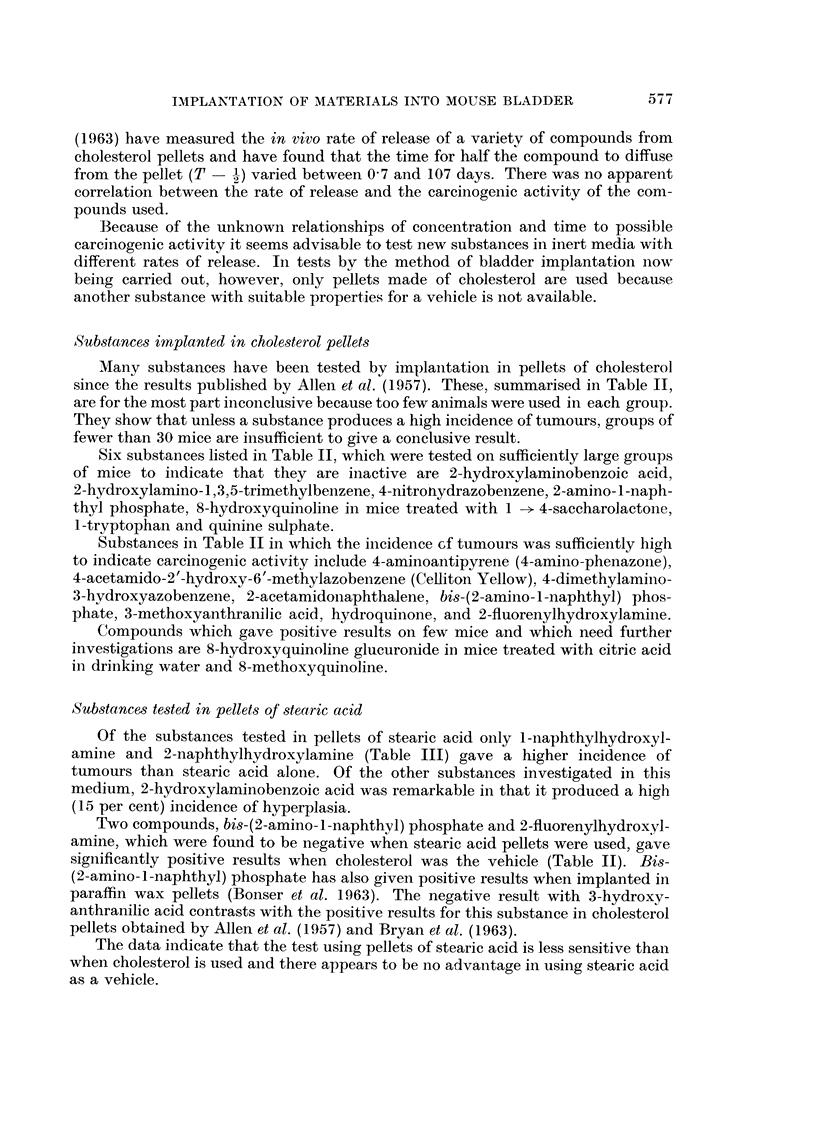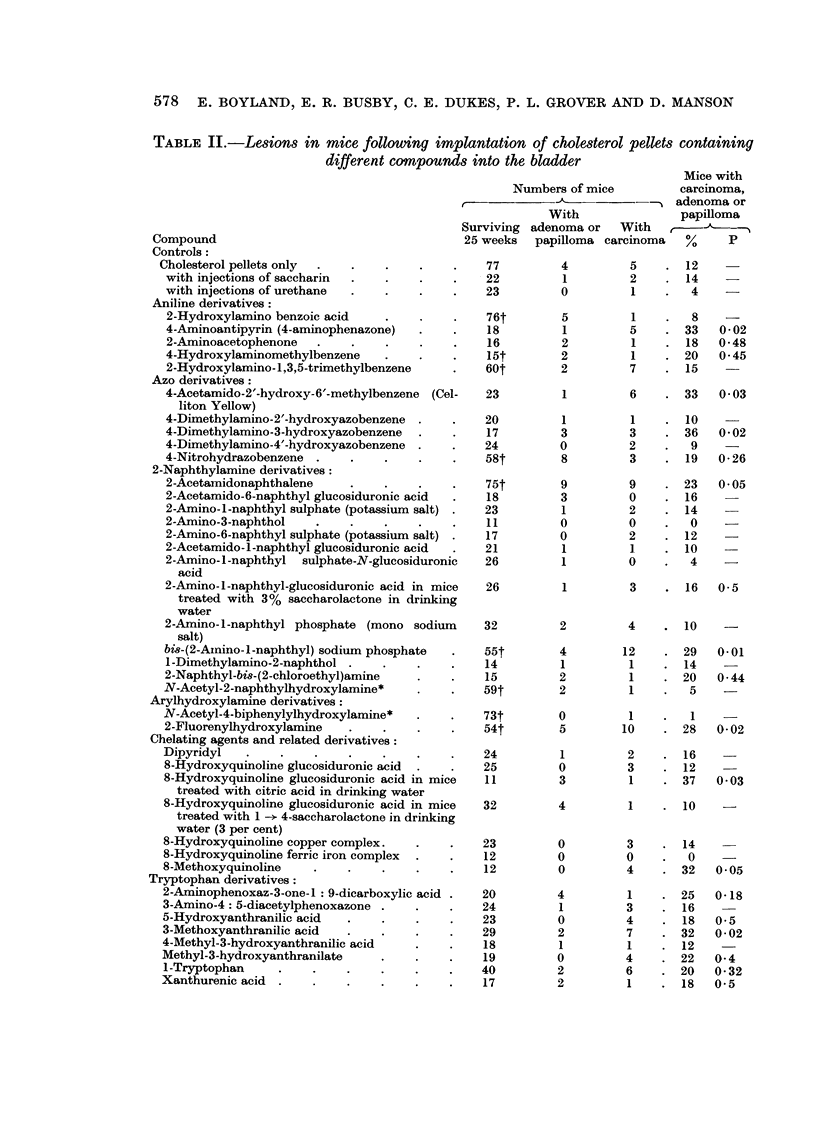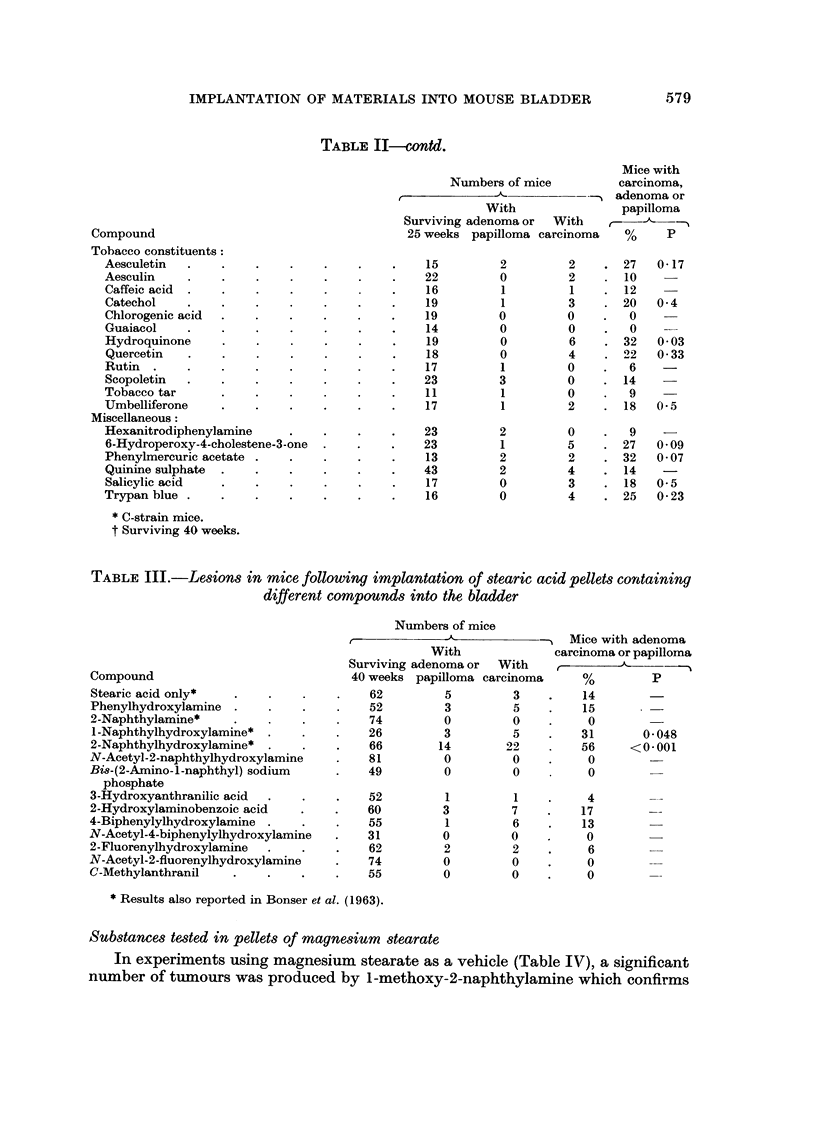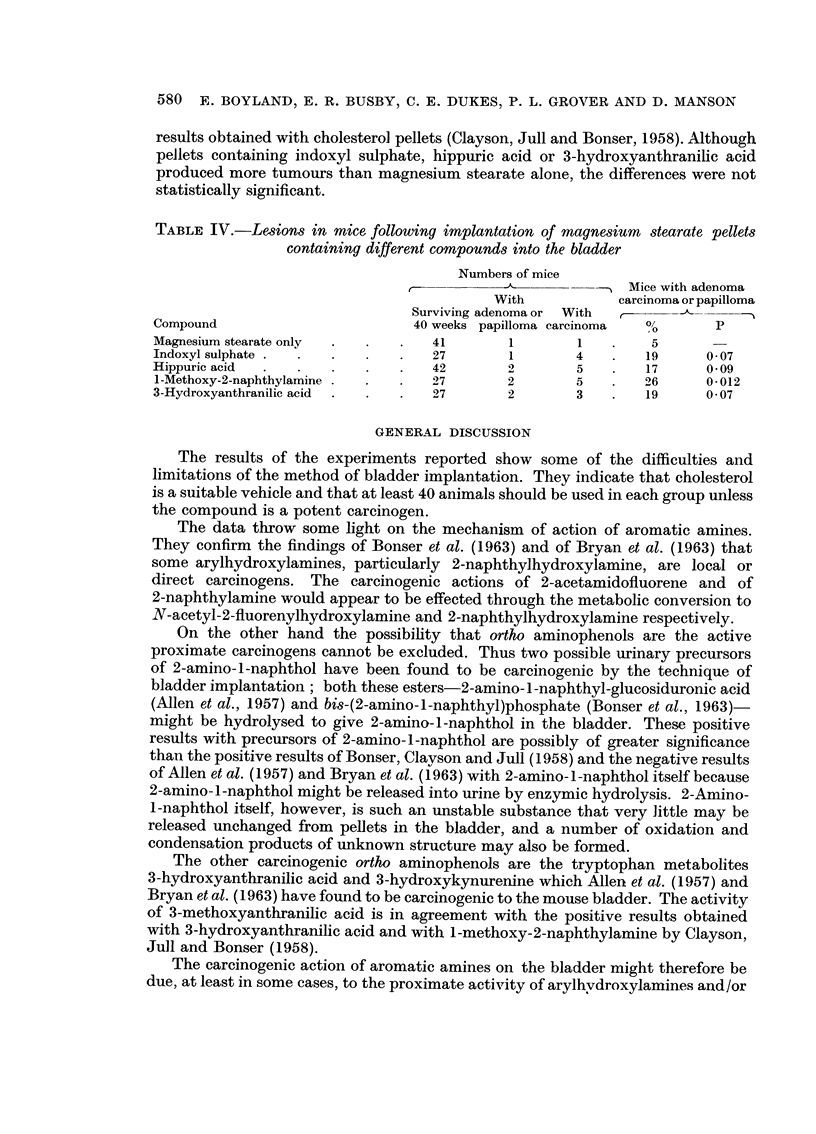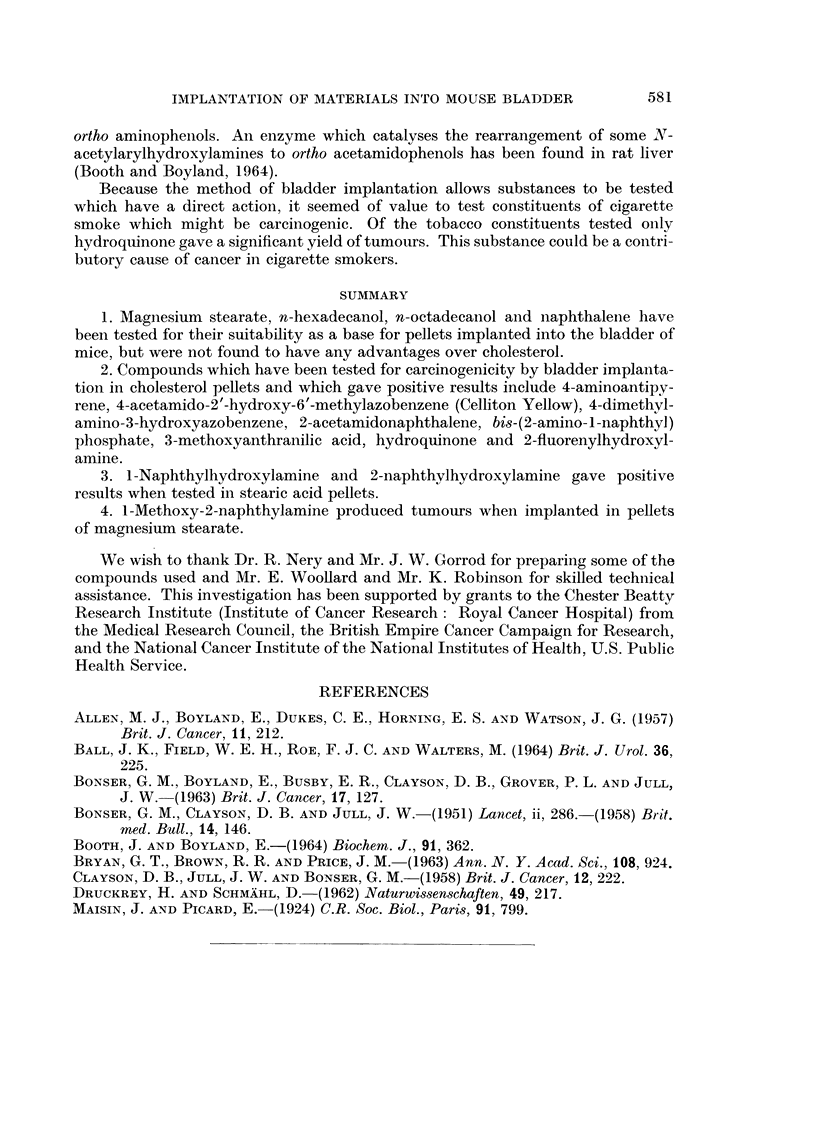# Further Experiments on Implantation of Materials into the Urinary Bladder of Mice

**DOI:** 10.1038/bjc.1964.66

**Published:** 1964-09

**Authors:** E. Boyland, E. R. Busby, C. E. Dukes, P. L. Grover, D. Manson


### FURTHER EXPERIMENTS ON IMPLANTATION OF MATERIALS INTO THE URINARY BLADDER OF MICE

E. BOYLAND, E. R. BUSBY*, C. E. DUKES, P. L. GROVER, AND D. MANSON

*From the Chester Beatty Research Institute, Institute of Cancer Research : Royal Cancer Hospital, Fulham Road, London, S.W.3*

Received for publication May 1, 1964

ALTHOUGH Maisin and Picard (1924) induced cancer of the bladder by implanting pellets containing tar into the bladders of rats, Bonser, Clayson and Jull (1951) developed the technique of bladder implantation in mice, which as modified by Allen, Boyland, Dukes, Horning and Watson (1957) has been widely used. The method can be employed to indicate whether a substance is a direct carcinogen or not, because there is less possibility of metabolic change occurring than with other routes of administration. A few of the results obtained by the technique in this laboratory since the publication of Allen *et al.* (1957) have been published (Bonser, Boyland, Busby, Clayson, Grover and Jull, 1963), but further results are presented in this paper.

One of the difficulties of the method is the choice of a suitable medium with which to mix the substances to be tested. Most apparently inert materials induce tumours in a proportion of mice and even the most active carcinogens do not induce cancer in all the mice treated. For this reason a few substances which would be expected to be biologically inert have been tried.

### METHODS AND MATERIALS

The technique used was the same as that described by Allen *et al.* (1957). With practice it is possible to expose the bladder and insert the pellet through a small incision in the skin. The incision may be so small that it can be closed with a single stitch. The probability that the incidence of tumours was due to chance was calculated by the $\chi^2$ test. Stock mice bred in the Chester Beatty Research Institute were used.

Cholesterol (Roche Products), and stearic acid (B.D.H.) were recrystallised from ethanol. Hexadeconal (cetyl alcohol) and octadecanol were purchased from British Drug Houses Ltd.

### RESULTS AND DISCUSSION

### *Tumour Induction with " Inert " Vehicles*

The incidence of tumours with inert substances some of which might be used in place of paraffin wax or cholesterol are given in Table I. Magnesium stearate gave a low incidence of tumours but it has the disadvantage that in the presence of chelating agents (*e.g. ortho* aminophenols and 8-hydroxyquinoline) the magnesium forms chelates. If such agents are being tested they would therefore be present as chelates.

* Present address—St. George's Hospital, London, S.W.1.

TABLE I.—*Incidence of bladder tumours in mice implanted with inert materials*

| | | Numbers of mice | | | |
|---|---|---|---|---|---|
| Substance | Reference | Surviving 30 weeks | With adenoma or papilloma | With carcinoma | Tumour incidence % |
| Cholesterol | Bryan *et al.*, 1963 | 87 | ? | 7 | 8 |
| Cholesterol | Allen *et al.*, 1957 | 21 | 0 | 1 | 5 |
| Cholesterol | Bonser *et al.*, 1958 | 55 | ? | 5 | 9 |
| Cholesterol | This paper | 77 | 4 | 5 | 12 |
| Stearic acid | Bonser *et al.*, 1963 | 62 | 5 | 3 | 13 |
| Paraffin wax | Bonser *et al.*, 1958 | 56 | ? | 2 | 4 |
| Paraffin wax | Bonser *et al.*, 1963 | 82 | 1 | 1 | 2 |
| Paraffin wax | Ball *et al.*, 1964 | 62 | | 22 | 32 |
| Smooth glass | ,, | 67 | | 3 | 4 |
| Roughened glass | ,, | 63 | | 18 | 29 |
| Magnesium stearate | This paper | 41 | 1 | 1 | 5 |
| *n*-Hexadecanol (cetyl alcohol) | This paper | 69 | 2 | 6 | 12 |
| *n*-Octadecanol | This paper | 50 | 7 | 6 | 26 |
| Naphthalene | This paper | 23 | 0 | 1 | 4 |

Two saturated alcohols were tested and found to have no advantages over cholesterol ; *n*-hexadecanol gave the same incidence of tumours as cholesterol (12 per cent) and *n*-octadecanol an even higher incidence (26 per cent). Naphthalene is a substance which appeared to have the requisite properties and is easily obtained in a pure condition. Although it gave a low incidence of tumours (4 per cent), the pellets rapidly disintegrated in the bladder. Such quick disintegration shortens the time during which the bladder is exposed to the substance under test.

Although Bonser, Clayson and Jull (1958) and Bonser, Boyland, Busby, Clayson, Grover and Jull (1963) obtained less than 5 per cent of tumours with paraffin wax, Ball, Field, Roe and Walters (1964) found a high incidence (32 per cent) of carcinomata in mice implanted with the same preparation of paraffin wax, and showed that the implantation of roughened glass beads induced a much higher incidence of tumours than did smooth glass beads.

The results of the present experiments and of other investigations show that all inert substances which have been tried induce a certain number of tumours. In the present work cholesterol, stearic acid and magnesium stearate have been used as vehicles for the tests. Both cholesterol and paraffin wax appear to be suitable vehicles, but cholesterol is a single chemical substance while paraffin wax is a mixture of hydrocarbons of variable composition.

Pellets made of stearic acid remain intact in the mouse bladder for only a few weeks whereas cholesterol pellets remain in the bladder for at least a year. This means that substances are released more rapidly from stearic acid than from cholesterol or paraffin wax. If the substance under test has a threshold concentration for carcinogenic action then it might be released too slowly from cholesterol to give an effective concentration. Thus Allen *et al.* (1957) showed that 3-hydroxyanthranilic acid, which was released very slowly from paraffin wax, was not carcinogenic in wax pellets but was when cholesterol pellets were used. On the other hand slower release would probably increase the carcinogenic action as the activity of other carcinogens (*e.g.* aminostilbenes) has been shown to be proportional to $CT^N$, where $C$ is the concentration of the carcinogen and $T$ the time of exposure and $N$ is a constant greater than 2 (Duckrey and Schmähl, 1962). Bryan, Brown and Price

(1963) have measured the *in vivo* rate of release of a variety of compounds from cholesterol pellets and have found that the time for half the compound to diffuse from the pellet ($T - \frac{1}{2}$) varied between 0·7 and 107 days. There was no apparent correlation between the rate of release and the carcinogenic activity of the compounds used.

Because of the unknown relationships of concentration and time to possible carcinogenic activity it seems advisable to test new substances in inert media with different rates of release. In tests by the method of bladder implantation now being carried out, however, only pellets made of cholesterol are used because another substance with suitable properties for a vehicle is not available.

*Substances implanted in cholesterol pellets*

Many substances have been tested by implantation in pellets of cholesterol since the results published by Allen *et al.* (1957). These, summarised in Table II, are for the most part inconclusive because too few animals were used in each group. They show that unless a substance produces a high incidence of tumours, groups of fewer than 30 mice are insufficient to give a conclusive result.

Six substances listed in Table II, which were tested on sufficiently large groups of mice to indicate that they are inactive are 2-hydroxylaminobenzoic acid, 2-hydroxylamino-1,3,5-trimethylbenzene, 4-nitrohydrazobenzene, 2-amino-1-naphthyl phosphate, 8-hydroxyquinoline in mice treated with 1 → 4-saccharolactone, 1-tryptophan and quinine sulphate.

Substances in Table II in which the incidence of tumours was sufficiently high to indicate carcinogenic activity include 4-aminoantipyrene (4-amino-phenazone), 4-acetamido-2′-hydroxy-6′-methylazobenzene (Celliton Yellow), 4-dimethylamino-3-hydroxyazobenzene, 2-acetamidonaphthalene, *bis*-(2-amino-1-naphthyl) phosphate, 3-methoxyanthranilic acid, hydroquinone, and 2-fluorenylhydroxylamine.

Compounds which gave positive results on few mice and which need further investigations are 8-hydroxyquinoline glucuronide in mice treated with citric acid in drinking water and 8-methoxyquinoline.

*Substances tested in pellets of stearic acid*

Of the substances tested in pellets of stearic acid only 1-naphthylhydroxylamine and 2-naphthylhydroxylamine (Table III) gave a higher incidence of tumours than stearic acid alone. Of the other substances investigated in this medium, 2-hydroxylaminobenzoic acid was remarkable in that it produced a high (15 per cent) incidence of hyperplasia.

Two compounds, *bis*-(2-amino-1-naphthyl) phosphate and 2-fluorenylhydroxylamine, which were found to be negative when stearic acid pellets were used, gave significantly positive results when cholesterol was the vehicle (Table II). *Bis*-(2-amino-1-naphthyl) phosphate has also given positive results when implanted in paraffin wax pellets (Bonser *et al.* 1963). The negative result with 3-hydroxyanthranilic acid contrasts with the positive results for this substance in cholesterol pellets obtained by Allen *et al.* (1957) and Bryan *et al.* (1963).

The data indicate that the test using pellets of stearic acid is less sensitive than when cholesterol is used and there appears to be no advantage in using stearic acid as a vehicle.

TABLE II.—*Lesions in mice following implantation of cholesterol pellets containing different compounds into the bladder*

| Compound | Numbers of mice: Surviving 25 weeks | Numbers of mice: With adenoma or papilloma | Numbers of mice: With carcinoma | Mice with carcinoma, adenoma or papilloma: % | Mice with carcinoma, adenoma or papilloma: P |
|---|---|---|---|---|---|
| Controls : | | | | | |
| Cholesterol pellets only | 77 | 4 | 5 | 12 | — |
| with injections of saccharin | 22 | 1 | 2 | 14 | — |
| with injections of urethane | 23 | 0 | 1 | 4 | — |
| Aniline derivatives : | | | | | |
| 2-Hydroxylamino benzoic acid | 76† | 5 | 1 | 8 | — |
| 4-Aminoantipyrin (4-aminophenazone) | 18 | 1 | 5 | 33 | 0·02 |
| 2-Aminoacetophenone | 16 | 2 | 1 | 18 | 0·48 |
| 4-Hydroxylaminomethylbenzene | 15† | 2 | 1 | 20 | 0·45 |
| 2-Hydroxylamino-1,3,5-trimethylbenzene | 60† | 2 | 7 | 15 | — |
| Azo derivatives : | | | | | |
| 4-Acetamido-2′-hydroxy-6′-methylbenzene (Celliton Yellow) | 23 | 1 | 6 | 33 | 0·03 |
| 4-Dimethylamino-2′-hydroxyazobenzene | 20 | 1 | 1 | 10 | — |
| 4-Dimethylamino-3-hydroxyazobenzene | 17 | 3 | 3 | 36 | 0·02 |
| 4-Dimethylamino-4′-hydroxyazobenzene | 24 | 0 | 2 | 9 | — |
| 4-Nitrohydrazobenzene | 58† | 8 | 3 | 19 | 0·26 |
| 2-Naphthylamine derivatives : | | | | | |
| 2-Acetamidonaphthalene | 75† | 9 | 9 | 23 | 0·05 |
| 2-Acetamido-6-naphthyl glucosiduronic acid | 18 | 3 | 0 | 16 | — |
| 2-Amino-1-naphthyl sulphate (potassium salt) | 23 | 1 | 2 | 14 | — |
| 2-Amino-3-naphthol | 11 | 0 | 0 | 0 | — |
| 2-Amino-6-naphthyl sulphate (potassium salt) | 17 | 0 | 2 | 12 | — |
| 2-Acetamido-1-naphthyl glucosiduronic acid | 21 | 1 | 1 | 10 | — |
| 2-Amino-1-naphthyl sulphate-*N*-glucosiduronic acid | 26 | 1 | 0 | 4 | — |
| 2-Amino-1-naphthyl-glucosiduronic acid in mice treated with 3% saccharolactone in drinking water | 26 | 1 | 3 | 16 | 0·5 |
| 2-Amino-1-naphthyl phosphate (mono sodium salt) | 32 | 2 | 4 | 10 | — |
| *bis*-(2-Amino-1-naphthyl) sodium phosphate | 55† | 4 | 12 | 29 | 0·01 |
| 1-Dimethylamino-2-naphthol | 14 | 1 | 1 | 14 | — |
| 2-Naphthyl-*bis*-(2-chloroethyl)amine | 15 | 2 | 1 | 20 | 0·44 |
| *N*-Acetyl-2-naphthylhydroxylamine* | 59† | 2 | 1 | 5 | — |
| Arylhydroxylamine derivatives : | | | | | |
| *N*-Acetyl-4-biphenylylhydroxylamine* | 73† | 0 | 1 | 1 | — |
| 2-Fluorenylhydroxylamine | 54† | 5 | 10 | 28 | 0·02 |
| Chelating agents and related derivatives : | | | | | |
| Dipyridyl | 24 | 1 | 2 | 16 | — |
| 8-Hydroxyquinoline glucosiduronic acid | 25 | 0 | 3 | 12 | — |
| 8-Hydroxyquinoline glucosiduronic acid in mice treated with citric acid in drinking water | 11 | 3 | 1 | 37 | 0·03 |
| 8-Hydroxyquinoline glucosiduronic acid in mice treated with 1 → 4-saccharolactone in drinking water (3 per cent) | 32 | 4 | 1 | 10 | — |
| 8-Hydroxyquinoline copper complex | 23 | 0 | 3 | 14 | — |
| 8-Hydroxyquinoline ferric iron complex | 12 | 0 | 0 | 0 | — |
| 8-Methoxyquinoline | 12 | 0 | 4 | 32 | 0·05 |
| Tryptophan derivatives : | | | | | |
| 2-Aminophenoxaz-3-one-1 : 9-dicarboxylic acid | 20 | 4 | 1 | 25 | 0·18 |
| 3-Amino-4 : 5-diacetylphenoxazone | 24 | 1 | 3 | 16 | — |
| 5-Hydroxyanthranilic acid | 23 | 0 | 4 | 18 | 0·5 |
| 3-Methoxyanthranilic acid | 29 | 2 | 7 | 32 | 0·02 |
| 4-Methyl-3-hydroxyanthranilic acid | 18 | 1 | 1 | 12 | — |
| Methyl-3-hydroxyanthranilate | 19 | 0 | 4 | 22 | 0·4 |
| 1-Tryptophan | 40 | 2 | 6 | 20 | 0·32 |
| Xanthurenic acid | 17 | 2 | 1 | 18 | 0·5 |

TABLE II—*contd.*

| Compound | Numbers of mice: Surviving 25 weeks | Numbers of mice: With adenoma or papilloma | Numbers of mice: With carcinoma | Mice with carcinoma, adenoma or papilloma: % | Mice with carcinoma, adenoma or papilloma: P |
|---|---|---|---|---|---|
| Tobacco constituents : | | | | | |
| Aesculetin | 15 | 2 | 2 | 27 | 0·17 |
| Aesculin | 22 | 0 | 2 | 10 | — |
| Caffeic acid | 16 | 1 | 1 | 12 | — |
| Catechol | 19 | 1 | 3 | 20 | 0·4 |
| Chlorogenic acid | 19 | 0 | 0 | 0 | — |
| Guaiacol | 14 | 0 | 0 | 0 | — |
| Hydroquinone | 19 | 0 | 6 | 32 | 0·03 |
| Quercetin | 18 | 0 | 4 | 22 | 0·33 |
| Rutin | 17 | 1 | 0 | 6 | — |
| Scopoletin | 23 | 3 | 0 | 14 | — |
| Tobacco tar | 11 | 1 | 0 | 9 | — |
| Umbelliferone | 17 | 1 | 2 | 18 | 0·5 |
| Miscellaneous : | | | | | |
| Hexanitrodiphenylamine | 23 | 2 | 0 | 9 | — |
| 6-Hydroperoxy-4-cholestene-3-one | 23 | 1 | 5 | 27 | 0·09 |
| Phenylmercuric acetate | 13 | 2 | 2 | 32 | 0·07 |
| Quinine sulphate | 43 | 2 | 4 | 14 | — |
| Salicylic acid | 17 | 0 | 3 | 18 | 0·5 |
| Trypan blue | 16 | 0 | 4 | 25 | 0·23 |

* C-strain mice.

† Surviving 40 weeks.

TABLE III.—*Lesions in mice following implantation of stearic acid pellets containing different compounds into the bladder*

| Compound | Numbers of mice: Surviving 40 weeks | Numbers of mice: With adenoma or papilloma | Numbers of mice: With carcinoma | Mice with adenoma carcinoma or papilloma: % | Mice with adenoma carcinoma or papilloma: P |
|---|---|---|---|---|---|
| Stearic acid only* | 62 | 5 | 3 | 14 | — |
| Phenylhydroxylamine | 52 | 3 | 5 | 15 | — |
| 2-Naphthylamine* | 74 | 0 | 0 | 0 | — |
| 1-Naphthylhydroxylamine* | 26 | 3 | 5 | 31 | 0·048 |
| 2-Naphthylhydroxylamine* | 66 | 14 | 22 | 56 | <0·001 |
| *N*-Acetyl-2-naphthylhydroxylamine | 81 | 0 | 0 | 0 | — |
| *Bis*-(2-Amino-1-naphthyl) sodium phosphate | 49 | 0 | 0 | 0 | — |
| 3-Hydroxyanthranilic acid | 52 | 1 | 1 | 4 | — |
| 2-Hydroxylaminobenzoic acid | 60 | 3 | 7 | 17 | — |
| 4-Biphenylylhydroxylamine | 55 | 1 | 6 | 13 | — |
| *N*-Acetyl-4-biphenylylhydroxylamine | 31 | 0 | 0 | 0 | — |
| 2-Fluorenylhydroxylamine | 62 | 2 | 2 | 6 | — |
| *N*-Acetyl-2-fluorenylhydroxylamine | 74 | 0 | 0 | 0 | — |
| *C*-Methylanthranil | 55 | 0 | 0 | 0 | — |

* Results also reported in Bonser *et al.* (1963).

*Substances tested in pellets of magnesium stearate*

In experiments using magnesium stearate as a vehicle (Table IV), a significant number of tumours was produced by 1-methoxy-2-naphthylamine which confirms results obtained with cholesterol pellets (Clayson, Jull and Bonser, 1958). Although pellets containing indoxyl sulphate, hippuric acid or 3-hydroxyanthranilic acid produced more tumours than magnesium stearate alone, the differences were not statistically significant.

TABLE IV.—*Lesions in mice following implantation of magnesium stearate pellets containing different compounds into the bladder*

| Compound | Numbers of mice: Surviving 40 weeks | Numbers of mice: With adenoma or papilloma | Numbers of mice: With carcinoma | Mice with adenoma carcinoma or papilloma: % | Mice with adenoma carcinoma or papilloma: P |
|---|---|---|---|---|---|
| Magnesium stearate only | 41 | 1 | 1 | 5 | — |
| Indoxyl sulphate | 27 | 1 | 4 | 19 | 0·07 |
| Hippuric acid | 42 | 2 | 5 | 17 | 0·09 |
| 1-Methoxy-2-naphthylamine | 27 | 2 | 5 | 26 | 0·012 |
| 3-Hydroxyanthranilic acid | 27 | 2 | 3 | 19 | 0·07 |

### GENERAL DISCUSSION

The results of the experiments reported show some of the difficulties and limitations of the method of bladder implantation. They indicate that cholesterol is a suitable vehicle and that at least 40 animals should be used in each group unless the compound is a potent carcinogen.

The data throw some light on the mechanism of action of aromatic amines. They confirm the findings of Bonser *et al.* (1963) and of Bryan *et al.* (1963) that some arylhydroxylamines, particularly 2-naphthylhydroxylamine, are local or direct carcinogens. The carcinogenic actions of 2-acetamidofluorene and of 2-naphthylamine would appear to be effected through the metabolic conversion to *N*-acetyl-2-fluorenylhydroxylamine and 2-naphthylhydroxylamine respectively.

On the other hand the possibility that *ortho* aminophenols are the active proximate carcinogens cannot be excluded. Thus two possible urinary precursors of 2-amino-1-naphthol have been found to be carcinogenic by the technique of bladder implantation ; both these esters—2-amino-1-naphthyl-glucosiduronic acid (Allen *et al.*, 1957) and *bis*-(2-amino-1-naphthyl)phosphate (Bonser *et al.*, 1963)—might be hydrolysed to give 2-amino-1-naphthol in the bladder. These positive results with precursors of 2-amino-1-naphthol are possibly of greater significance than the positive results of Bonser, Clayson and Jull (1958) and the negative results of Allen *et al.* (1957) and Bryan *et al.* (1963) with 2-amino-1-naphthol itself because 2-amino-1-naphthol might be released into urine by enzymic hydrolysis. 2-Amino-1-naphthol itself, however, is such an unstable substance that very little may be released unchanged from pellets in the bladder, and a number of oxidation and condensation products of unknown structure may also be formed.

The other carcinogenic *ortho* aminophenols are the tryptophan metabolites 3-hydroxyanthranilic acid and 3-hydroxykynurenine which Allen *et al.* (1957) and Bryan *et al.* (1963) have found to be carcinogenic to the mouse bladder. The activity of 3-methoxyanthranilic acid is in agreement with the positive results obtained with 3-hydroxyanthranilic acid and with 1-methoxy-2-naphthylamine by Clayson, Jull and Bonser (1958).

The carcinogenic action of aromatic amines on the bladder might therefore be due, at least in some cases, to the proximate activity of arylhydroxylamines and/or

*ortho* aminophenols. An enzyme which catalyses the rearrangement of some *N*-acetylarylhydroxylamines to *ortho* acetamidophenols has been found in rat liver (Booth and Boyland, 1964).

Because the method of bladder implantation allows substances to be tested which have a direct action, it seemed of value to test constituents of cigarette smoke which might be carcinogenic. Of the tobacco constituents tested only hydroquinone gave a significant yield of tumours. This substance could be a contributory cause of cancer in cigarette smokers.

### SUMMARY

1. Magnesium stearate, *n*-hexadecanol, *n*-octadecanol and naphthalene have been tested for their suitability as a base for pellets implanted into the bladder of mice, but were not found to have any advantages over cholesterol.

2. Compounds which have been tested for carcinogenicity by bladder implantation in cholesterol pellets and which gave positive results include 4-aminoantipyrene, 4-acetamido-2′-hydroxy-6′-methylazobenzene (Celliton Yellow), 4-dimethylamino-3-hydroxyazobenzene, 2-acetamidonaphthalene, *bis*-(2-amino-1-naphthyl) phosphate, 3-methoxyanthranilic acid, hydroquinone and 2-fluorenylhydroxylamine.

3. 1-Naphthylhydroxylamine and 2-naphthylhydroxylamine gave positive results when tested in stearic acid pellets.

4. 1-Methoxy-2-naphthylamine produced tumours when implanted in pellets of magnesium stearate.

We wish to thank Dr. R. Nery and Mr. J. W. Gorrod for preparing some of the compounds used and Mr. E. Woollard and Mr. K. Robinson for skilled technical assistance. This investigation has been supported by grants to the Chester Beatty Research Institute (Institute of Cancer Research : Royal Cancer Hospital) from the Medical Research Council, the British Empire Cancer Campaign for Research, and the National Cancer Institute of the National Institutes of Health, U.S. Public Health Service.

### REFERENCES

ALLEN, M. J., BOYLAND, E., DUKES, C. E., HORNING, E. S. AND WATSON, J. G. (1957) *Brit. J. Cancer*, **11**, 212.

BALL, J. K., FIELD, W. E. H., ROE, F. J. C. AND WALTERS, M. (1964) *Brit. J. Urol.* **36**, 225.

BONSER, G. M., BOYLAND, E., BUSBY, E. R., CLAYSON, D. B., GROVER, P. L. AND JULL, J. W.—(1963) *Brit. J. Cancer*, **17**, 127.

BONSER, G. M., CLAYSON, D. B. AND JULL, J. W.—(1951) *Lancet*, ii, 286.—(1958) *Brit. med. Bull.*, **14**, 146.

BOOTH, J. AND BOYLAND, E.—(1964) *Biochem. J.*, **91**, 362.

BRYAN, G. T., BROWN, R. R. AND PRICE, J. M.—(1963) *Ann. N. Y. Acad. Sci.*, **108**, 924.

CLAYSON, D. B., JULL, J. W. AND BONSER, G. M.—(1958) *Brit. J. Cancer*, **12**, 222.

DRUCKREY, H. AND SCHMÄHL, D.—(1962) *Naturwissenschaften*, **49**, 217.

MAISIN, J. AND PICARD, E.—(1924) *C.R. Soc. Biol., Paris*, **91**, 799.